# Comprehensive Characterization of Five Lactococcus Strains: From Phenotypic Traits to Genomic Features

**DOI:** 10.32607/actanaturae.27747

**Published:** 2025

**Authors:** I. D. Antipenko, N. P. Sorokina, I. V. Kucherenko, E. V. Kuraeva, E. S. Masezhnaya, M. Yu. Shkurnikov

**Affiliations:** Laboratory for Research on Molecular Mechanisms of Longevity, Department of Biology and Biotechnology, HSE University, Moscow, 101000 Russia; All-Russian Research Institute of Butter and Cheese Making, Branch of the Gorbatov Federal Research Center for Food Systems, Uglich, 109316 Russia

**Keywords:** Lactococcus, fermentation, genomic profiling, prophages, starter cultures, bacteriophages

## Abstract

The efficiency of dairy product fermentation directly depends on the properties of the lactic acid bacteria used, particularly on their metabolic activity and resistance to bacteriophages. Therefore, an understanding of the relationships between the genetic and phenotypic traits of industrial strains is of elevated importance. In this study, we comprehensively analyzed five Lactococcus strains widely used in the Russian dairy industry, combining whole-genome sequencing with phenotypic profiling. Despite the fact of genetic similarity among four of the L. lactis strains, we still identified significant differences in their metabolic activity. Comparative structural analysis of previously published genomes of 337 L. lactis and 147 L. cremoris strains revealed species-specific features of the lactose metabolism; in particular, the absence of the lacZ gene in L. cremoris. Notably, prophages were found in three of the studied strains, which was in correlation with their reduced acidification activity. L. lactis FNCPS 51n and 73n strains displayed resistance to all 50 tested bacteriophages, which may be associated with the presence of the AbiB abortive infection system. These findings underscore the importance of integrating genomic and phenotypic analyses when selecting efficient and phage-resistant Lactococcus starters in the dairy industry.

## INTRODUCTION

Members of the genus Lactococcus, which are able to efficiently convert lactose into lactic acid, are among the key microorganisms used in the production of fermented dairy products [1].

In the dairy industry, the most widely used cultures are L. lactis and L. cremoris, with the latter having been recently elevated to the species level [2]. These species differ in the genes associated with carbohydrate and amino acid metabolism [3], as well as in their stress response mechanisms [4].

I addition, there are industrially significant differences at the intraspecific level. For example, the subspecies L. lactis subsp. lactis comprises the diacetylactis biovar that is capable of metabolizing citrate into diacetyl, a compound that imparts a characteristic buttery, creamy flavor to the product [5], which is an important component of the aroma of cheeses like Camembert, Emmental, and Cheddar [6]. Fermentation strain combinations are selected based on the product type: diacetylactis and L. cremoris are frequently used in fermented milk production, whereas L. lactis subsp. lactis is used in cheesemaking [7]. In the case of lactococcal starters, the fermentation rate has been shown to depend more on the individual characteristics of the strain than on its species classification [8].

The diversity of technologically significant traits in Lactococcus has expanded through evolutionary processes and horizontal gene transfer, including plasmids carrying genes associated with sugar metabolism, flavor compound synthesis, and bacteriophage resistance [9]. Different countries and regions use both commercial and local lactic acid bacteria (LAB) strains selected based on either a long tradition of their use or their unique technological properties [10].

In this study, we comparatively analyzed five Lactococcus strains widely used in Russia in the production of fermented dairy products. We also identified the metabolic and genetic characteristics of these strains, including resistance to bacteriophages, and identified the traits underlying their technological value.

## EXPERIMENTAL

**Strains and culturing conditions**

In this study, we used five strains: L. cremoris FNCPS 23 (GCA_044990555.1) and L. lactis FNCPS 51_n, 43_n, 81_n, and 73_n (GCA_044990575.1, GC A _ 0 4 4 9 9 0 5 3 5 . 1 , GC A _ 0 4 4 9 9 0 6 0 5 . 1 , a n d GCA_044990625.1, respectively) from the collection of the All-Russian Research Institute of Butter and Cheese Making (VNIIMS, a branch of the Gorbatov Federal Research Center for Food Systems of the Russian Academy of Sciences). Strains 81_n and 43_n were isolated from indigenous sour cream, and the others were isolated from milk. All the strains were isolated from products from the Yaroslavl region of Russia, except the L. cremoris FNCPS 23 strain, which was isolated from a sample from Lithuania.

**Phenotypic characterization**

Growth was assessed spectrophotometrically using a KFK-3-ZOMZ photoelectric photometer (Zagorsk Optical and Mechanical Plant, Russia). Strains were cultured in 10% sterile reconstituted skim milk added with a 1% inoculum from 16-h culture at 30°C. The optical density (OD) was measured at 560 nm at 60-min intervals for 10 h

To determine the maximum titratable acidity, 10% sterile reconstituted skim milk was combined with 0.1% of a 16-h lactococcal culture and incubated at 30 ± 1°C for 7 days. Acidity was evaluated by titration with a NaOH solution using a Titrette bottletop burette, 50 mL, (Brand, Germany) according to a previously described method [11] and expressed as Therner degrees (°T).

The pH was measured in 10% sterile reconstituted skim milk before fermentation (control pH, 6.53) and at control points during fermentation using a STARTER 2100 digital pH meter (Ohaus, Switzerland).

Coagulation activity was assessed by the ability of the strain to form a clot in 10% reconstituted skim milk containing 0.015% litmus. For this purpose, sterile litmus milk was combined with an inoculum of each strain in three replicates. Clot formation and litmus color changes (reduction) were assessed hourly during incubation at 30 and 40°C [12].

Acetoin and diacetyl formation was determined using the Voges-Proskauer test: a 48-h culture was mixed with 30% KOH, and color intensity was assessed on a 5-point scale.

**Analysis of bacteriophage lytic profiles**

The lytic spectrum of bacteriophages was determined by culturing them on double-layer agar in Petri dishes [13]. Sensitivity to bacteriophages was assessed by the presence or absence of zones of clearing at the point of phage spotting.

**Genome sequencing**

DNA for genome sequencing was isolated using an ExtractDNA Blood and Cells kit (Eurogen, Russia), according to the manufacturer’s instructions. DNA libraries were prepared using the MGIEasy Fast FS DNA Library Prep Set V2.0 (Cat. No. 940-001196-00, MGI) according to the manufacturer’s protocol. Library quality was assessed using a Qubit 1X dsDNA High Sensitivity DNA Assay kit (Cat. No. Q33230, Thermo Fisher Scientific, USA) and a Qubit Fluorometer (Thermo Fisher Scientific, USA). The length of the DNA library fragments was estimated by QIAxcel Advanced capillary gel electrophoresis using a QX DNA Fast Analysis kit (Cat. No. 929008, Qiagen). Sequencing was performed using an FCS flow cell on an MGI DNBSEQ-G50 platform (BGI, China) in PE150 mode.

Bacterial genomes were assembled using SPAdes [14] in isolate mode. To improve the quality of the final assembly, raw reads were aligned to contigs using Bowtie2 [15], after which the alignment files were sorted and indexed using SAMtools [16] then transferred to Pilon [17] to correct assembly inaccuracies. Assembly quality was assessed using QUAST [18], and the completeness of the assembled genomes was evaluated using BUSCO [19].

**Genome analysis**

Genome annotation and functional analysis were performed using the NCBI Prokaryotic Genome Annotation Pipeline [20], BlastKOALA [21], and the BV-BRC platform [22]. The presence of metabolic genes in the genomes of the Lactococcus strains was analyzed using the BV-BRC platform, based on high-quality, open-access complete genome assemblies: L. lactis (n = 337) and L. cremoris (n = 147).

Prophages in bacterial genomes were identified using the PHASTEST tool, with deep search settings [23]. Bacterial defense systems were identified using the DefenseFinder web tool (v2.0.0, model database v2.0.2) [24]. HMMER-based results were filtered using the following criteria: i-evalue ≤ 1e-5, profile coverage ≥ 70%, and sequence coverage ≥ 70%.

Additionally, spacers and CRISPR-Cas system components were identified using CRISPRCasFinder [25]. Plasmids were identified using PlasmidFinder v2.0.1 (database: 2020-07-13) with ≥ 95% identity and ≥ 60% coverage thresholds [26].

**Phylogenetic analysis**

Phylogenetic identification and determination of closely related strains was performed using tetranucleotide correlation analysis via the JSpeciesWS web service [27]. Average nucleotide identity (ANI) comparison was performed using the OrthoANI algorithm [28].

## RESULTS

**General genomic characteristics**

**Fig. 1:**
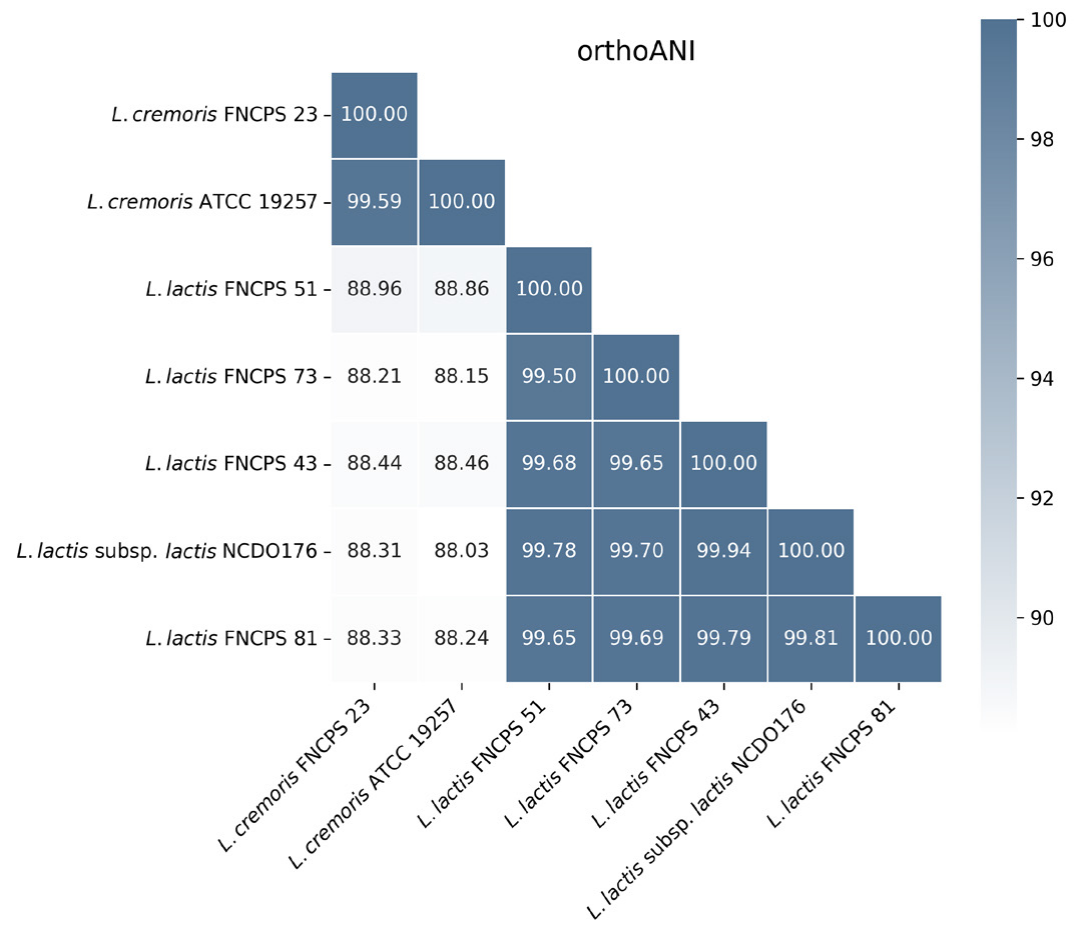
Heatmap of average nucleotide identity of orthologous genes (orthoANI). Numbers depict the percentage of average nucleotide identity among orthologous genes in different strains. Diagonal numbers (100%) indicate strain self-identity

Genomic sequence characteristics of the five analyzed strains are presented in Table 1. They proved similar to the reported characteristics of the strains of the corresponding LAB species. Four strains were classified as L. lactis, and one strain was classified as L. cremoris. The closest type-specific genomes were L. lactis subsp. lactis NCDO176 (Z-score > 0.996) for L. lactis and L. cremoris ATCC 19257 (Z-score = 0.998) for L. cremoris. OrthoANI analysis revealed high genome similarity among L. lactis strains (> 99.5%), indicating their close relation (Fig. 1). The L. cremoris strain was characterized by low ANI values (88 to 89%) compared with those of L. lactis, confirming its separate species status.

**Table 1:** General genome characteristics of the study Lactococcus strains

Parameter	L. lactis FNCPS 73n	L. lactis FNCPS 51n	L. lactis FNCPS 43n	L. lactis FNCPS 81n	L. cremoris FNCPS 23
Genome size, bp	3110896	2258993	3084214	2963565	2528857
GC-composition, %	34.91	35.52	34.97	35.35	35.78
tRNA	75	44	57	60	53
rRNA	8	5	7	8	5
Hypothetical proteins	965	720	907	1121	765
Proteins with established function	3349	2575	3292	3649	2224
CRISPR locus	-	1	1	-	9
Cas protein	-	-	-	-	-
Prophages	-	+	+	-	+
Plasmids	repUS33	repUS4, rep32	repUS33, repUS4	repUS33	repUS33

**Strain phenotyping**

**Fig. 2:**
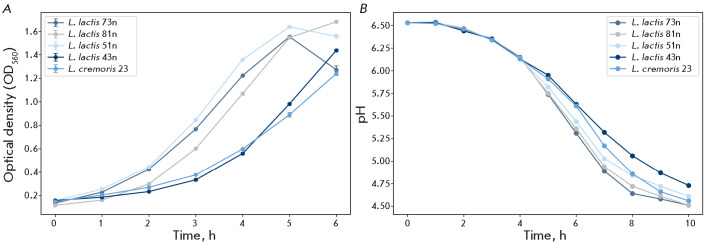
(A) Optical density changes in the culture medium during growth of the study strains. (B) pH changes in the culture medium during growth of the same strains

The results of the phenotypic tests of the study strains are presented in Table 2.

**Table 2:** Phenotypic characteristics of the study strains

Strain	Coagulation activity				Limiting acidity, °T	Diacetyl formation, points
	at 30°C, h		at 40°C, h			
	Litmus reduction	Coagulation	Litmus reduction	Coagulation		
L. lactis 73n	8	8	8	8	98	-
L. lactis 81n	6	9	6	11	96	-
L. lactis 51n	10	16	9	11	98	4/5
L. lactis 43n	12 incomplete	12	13	13	90	5/5
L. cremoris 23	13	14	-	-	88	-

Growth of the study strains was assessed by changes in optical density (OD) during culturing. The L. lactis 73n, 81n, and 51n strains demonstrated similar growth rates, whereas L. lactis 43n and L. cremoris 23 grew more slowly. Seven hours after the start of the experiment, OD reached a plateau, which may indicate the end of the active growth phase (Fig. 2A).

Differences among the strains were also evident in their limiting acidity: L. lactis 43n and L. cremoris 23 had the lowest values (Table 2). A similar pattern was observed in pH measurements: the steepest decrease in the pH of the culture medium was observed for L. lactis 73n and 81n, whereas minimal medium acidification by the final stage of culturing was observed for L. lactis 43n (Fig. 2B).

Assessment of coagulation activity at 30 and 40°C (Table 2) confirmed a high metabolic activity for L. lactis 73n and 81n: litmus reduction occurred within 6–8 h, and milk coagulation occurred within 8–11 h at both temperatures. L. lactis 51n demonstrated a temperature dependence: coagulation occurred 5 h earlier at 40°C than at 30°C. L. lactis 43n and L. cremoris 23 exhibited the least activity: coagulation occurred after 12–14 h, with 43n exhibiting incomplete litmus reduction. L. cremoris 23 did not exhibit coagulation or litmus reduction at 40°C.

**Metabolic gene analysis**

Proteolytic system and amino acid catabolism. Efficient growth of LAB in milk requires the breakdown of proteins, particularly casein that accounts for approximately 80% of all milk proteins [29]. The casein molecule is enriched in proline residues, which makes it accessible to caseinolytic proteases. Two types of extracellular proteinases, PI and PIII, have been described in Lactococcus. They differ in their specificity to casein fractions [29]. Lactosepin I (PI) preferentially hydrolyzes β-casein, forming over 100 oligopeptides of 4 to 30 amino acid residues in length. PIII exhibits a broader specificity, cleaving αs1-, β-, and κ-casein. However, half of industrial L. lactis strains lack the prtP gene encoding these enzymes [30].

The prtP gene was found in three of the five strains studied: L. cremoris 23 and L. lactis 51n and 81n. The proteins of L. cremoris 23 and L. lactis 51n showed high identity (97%), whereas a 427 amino acid deletion was found in L. lactis 81n, which reduced identity to 76.6–77.2%. All proteins are classified as PI-type proteinases. Comparison with the reference PI-type proteinase (PrtP, P16271) revealed a degree of identity of 97.8% in L. cremoris 23, 95.9% in L. lactis 51n, and 74.9% in L. lactis 81n.

**Fig. 3:**
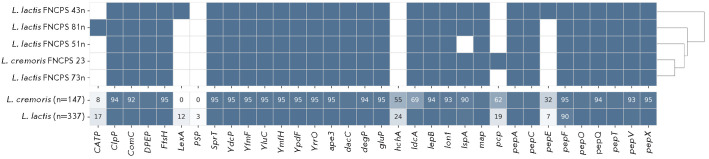
Presence of proteolytic enzyme genes in L. lactis (n = 337), L. cremoris (n = 147), and the five study strains. The upper panel shows a binary matrix: blue indicates the presence of a gene, and white denotes absence. The lower panel displays the percentage of each gene in the population; numerical values are shown only for genes present in less than 95% of the strains. Strains are grouped according to the similarity of their proteolytic genomic profiles; clustering results are presented as a dendrogram

Peptides formed during casein digestion are transported into the cell by the Opp, DtpT, and Dpp systems [31], whose genes are present in all five strains. In the cytoplasm, the peptides are cleaved by exoand endoproteases [29]. Comparative analysis of the genomes revealed a similar protease gene profile in all five strains, which was typical of the general pattern for the genus Lactococcus (Fig. 3).

The main differences between L. cremoris and L. lactis resided in the frequency of occurrence of the pyrrolidone carboxylate peptidase (pcp, [EC 3.4.19.3]) and peptidase E (pepE) genes that, according to the UniProt database, are localized in plasmids. The pcp gene was found in 62% of L. cremoris strains and only 19% of L. lactis strains, whereas pepE was found in 32% of L. cremoris strains and 7% of L. lactis strains. In contrast, the CATP (a protein of the CAAX aminoterminal protease family) and LexA genes were more common in L. lactis (17 and 12%, respectively) than in L. cremoris (8 and 0%, respectively).

The analyzed strains differed in the presence of five proteolytic enzyme genes. Namely, the L. cremoris 23 strain contained the pcp gene, the L. lactis 81n strain contained CATP, the L. lactis 43n strain contained LexA and pepE, and the 51n strain lacked the lspA gene encoding signal lipoprotein peptidase.

**Fig. 4:**
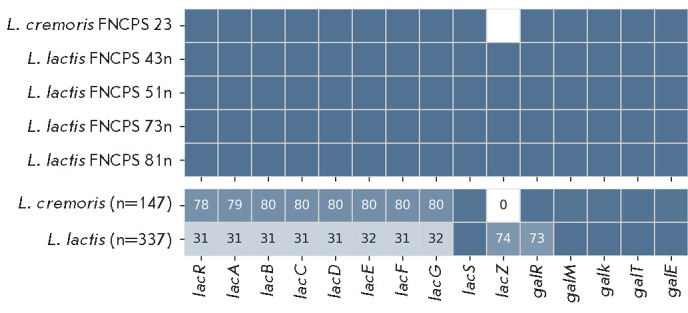
Presence of lactose metabolism genes in L. lactis (n = 337), L. cremoris (n = 147), and the five study strains. The upper panel shows a binary matrix: blue indicates the presence of a gene, and white indicates absence. The lower panel displays the percentage of each gene in the population; numerical values are shown only for genes present in less than 95% of the strains

Lactose metabolism. Lactose is the main carbon source for LAB in milk. Lactose and galactose are metabolized via the Leloir and tagatose-6-phosphate pathways [32]. Analysis of L. lactis strains revealed that 31% (104 of 337) of them contained all the genes of the tagatose-6-phosphate pathway, in contrast to 78% (115 of 147) for the L. cremoris strains. All the five studied strains also contained a complete set of the corresponding genes (Fig. 4).

The key enzyme of the Leloir pathway is β-galactosidase LacZ, which catalyzes the breakdown of lactose into glucose and galactose. The lacZ gene was not found in any of the L. cremoris genomes, including the strain FNCPS 23, but was identified in 74% of the L. lactis strains, including all four studied strains of this species (Fig. 4).

**Fig. 5:**
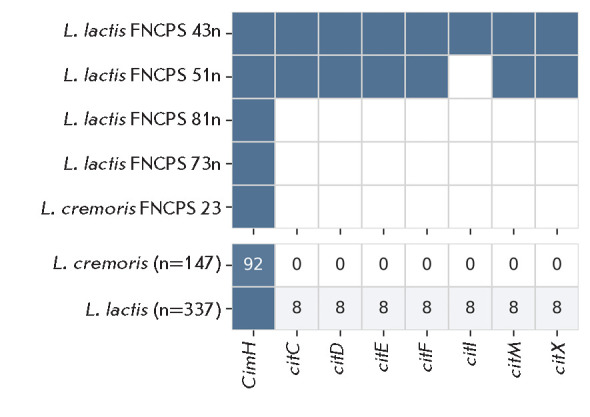
Presence of citrate metabolism genes in L. lactis (n = 337), L. cremoris (n = 147), and the five study strains. The upper panel shows a binary matrix: blue indicates the presence of a gene, and white indicates absence. The lower panel displays the percentage of each gene in the population; numerical values are shown only for genes present in less than 95% of the strains

Citrate metabolism. Among the study strains, only L. lactis 43n and 51n produced diacetyl, with 43n exhibiting higher activity (Table 2). Genetic analysis identified the genes responsible for citrate metabolism exclusively in these two strains (Fig. 5). All L. cremoris representatives lacked the corresponding genes: overall, they were found in only 8% (28 of 337) of the L. lactis strains, indicating a limited distribution of the diacetylactis biovar

**Bacteriophage resistance**

Bacteriophages are a common cause of mishaps in dairy product fermentation, leading to economic losses [33]. DNA of phages specific to Lactococcus and Streptococcus was found in 37% of samples of milk for fermentation in [34], which makes assessment of phage resistance an important step in the selection of industrial strains.

**Fig. 6:**
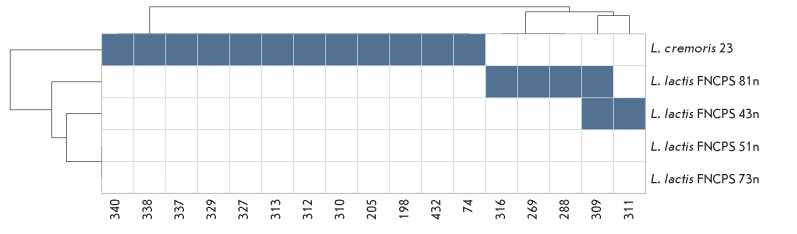
Lytic spectrum of bacteriophage interactions with the study strains. Bacteriophage names are shown on the X-axis. Blue cells indicate strain sensitivity to the corresponding phage, and white cells indicate lack of the lytic effect. Strains and bacteriophages are grouped according to the similarity of their lytic profiles; clustering results are presented as a dendrogram

Figure 6 shows the results of a test of 50 bacteriophages (only those that caused lysis of at least one strain are shown). The L. lactis 73n and 51n strains demonstrated resistance to all phages. Strains 81n and 43n and L. cremoris 23 were sensitive to some bacteriophages, but they retained resistance to others.

**Fig. 7:**
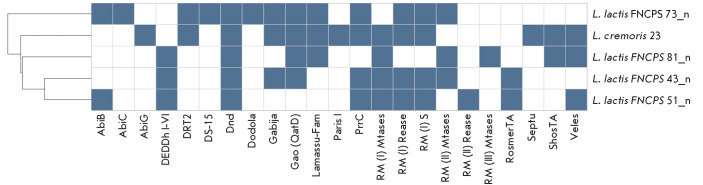
Profile of the bacterial immune systems in the study strains. Defense system names are shown on the X-axis. The study strains are depicted on the Y-axis. Blue cells indicate the presence of corresponding genes, and white cells indicate absence. Strains are grouped according to similarity in the abundance of defense system genes; clustering results are displayed as a dendrogram

Since differences in phage resistance may be associated with variability in the phage-specific defense systems encoded by bacterial genomes, we conducted our analysis using DefenseFinder (Fig. 7). The only system common to all strains was Dnd, an innate immunity mechanism based on DNA phosphorothioate modifications [35].

The AbiB system was detected only in the resistant strains. In addition, AbiC, DS-15, and Dodola were found in L. lactis 73n. A unique type II restrictionmodification (RM) system was identified in L. lactis 51n and was lacking in the other strains. The L. lactis 81n strain was the most sensitive to phage infection (4/50 phages caused lysis). This strain lacked a type I RM system restriction enzyme and S subunit genes, as well as PrrC system genes, which may explain its sensitivity to phages. L. cremoris 23 demonstrated the highest sensitivity, being lysed by 12 phages that were inactive against other strains. In this case, no defense system present in all L. lactis strains but absent in L. cremoris 23 was detected, which may indicate the involvement of the cell wall structure in susceptibility to phage infection [36].

Cas (types I–IV) genes were not detected, but CRISPR spacers were present in the genomes of all strains, except L. lactis 81n and 73n.

**Prophage detection**

Genome analysis revealed phage nucleotide sequences, indicating the presence of prophages in most of the study strains. No phage sequence insertions were detected in the L. lactis 73n and 81n strains, which indicates the absence of integrated prophages.

Two contigs with phage genes were found in the L. cremoris 23 genome: one (33.5 kb) is similar to PHAGE_Lactoc_62503, and the other (8.1 kb) is similar to PHAGE_Lactoc_bIL309. These contigs contain 40 and 11 coding sequences with a GC content of 35.04–35.29%. The first fragment is likely a functional prophage, whereas the second is a residual inactive insertion.

The L. lactis 51n genome includes four contigs with phage sequences (2.8–7.3 kb, a total of about 22 kb) which are similar to the phages bIL312, bIL309, D4410, and bIL286. These regions may be either vestigial remnants or parts of a single prophage separated during genome assembly.

In L. lactis 43n, we found six phage-containing contigs, with the largest ones reaching 13.5 kb (a total length of approximately 44 kb). We identified sequences similar to the phages bIL312, bIL285, and bIL309, as well as 98201, bIL311, and BK5_T. Phage proteins are synthesized from both DNA strands, which indicates the double-stranded nature of the phage genome.

Since none of the phage DNA fragments in the L. lactis 43n and L. lactis 51n strains were integrated into the contig containing bacterial genes, we suggest that the identified sequences are DNA of phages that entered the sample during the culture stage, rather than prophages integrated into the genome.

## DISCUSSION

In this study, we characterized five industrial strains of the genus Lactococcus. Genomic analysis revealed high similarity among the L. lactis strains, indicating their clonal origin, whereas L. cremoris 23 proved taxonomically distinct. Despite their close relation, the strains differed in their metabolic activities: L. lactis 73n and 81n were characterized by high growth and acid production rates, whereas L. lactis 43n and L. cremoris 23 were characterized by low growth and acid production rates. Strain 51n, which exhibited moderate activity, increased the rate of milk coagulation at 40°C, a temperature unusual for Lactococcus [37], which may be important for the production of cooked cheeses (Parmesan, Emmental, etc.). In addition, the L. lactis strains 43n and 51n happened to belong to the diacetylactis biovar, which was confirmed by the presence of citrate metabolism genes and the ability to produce diacetyl. In this case, the level of diacetyl production was lower in strain 51n than in 43n, which is probably due to the lack of the citrate lyase transcriptional regulator gene (citI) that is involved in the activation of the transcription of the corresponding operon in the presence of citrate [38]. The correlation between diacetyl production activity and the presence of the citI gene may be of industrial interest; however, experiments on genome editing and assessment of the mRNA expression of the corresponding genes are necessary to confirm its role.

Genomic analysis revealed genes encoding the Opp, DtpT, and Dpp peptide transport systems, as well as a characteristic set of LAB proteolytic enzymes [29]. The presence of the prtP gene encoding type I extracellular protease in the L. lactis 51n and L. cremoris 23 strains did not correlate with their metabolic activity. Recently, the abundance of individual peptidase genes (Pcp, PepE/G, PepI, PepR, PepL, and PepQ) in lactic acid bacteria genomes has been shown to vary significantly, affecting fermentation activity [39]. The study strains also differed in the composition of the genes encoding individual intracellular peptidases, including pcp, pepE, CATP, lspA, and lexA. Although the identified variations did not explain the phenotypic differences, they may have functional significance. To confirm their role in phenotype formation, functional testing of the corresponding genes and assessment of proteolytic activity are necessary. Furthermore, the lack of an obvious correlation may be due to differences in the copy numbers of these genes and their possible plasmid localization, which has not been assessed in this study and constitutes a limitation.

We confirmed significant differences in lactose metabolism between L. lactis and L. cremoris, as previously noted in the literature [1, 40]. The absence of lacZ in L. cremoris is a species-specific feature, whereas this gene is present in most L. lactis. In this case, 80% of L. cremoris and one-third of L. lactis express genes for the tagatose-6-phosphate pathway. All the L. lactis strains studied possess genes for both pathways, which reflects their adaptation to industrial conditions.

The L. lactis 73n and 51n strains demonstrated resistance to all the phages tested. These strains are characterized by the presence of the AbiB abortive infection system, but total resistance is likely due to a number of factors. In addition, as shown previously, the AbiB system is effective primarily against Lactococcus 936-type phages [41]. Therefore, to confirm its contribution to the resistance of these strains, it is necessary to identify the types of phages used in testing. Our results are consistent with the findings of longitudinal monitoring of phage dynamics in cheese-making factories where lactococcal resistance to bacteriophages was also associated with abortive infection systems (Abi) [42]. It was discovered that starter culture rotation affected the composition and abundance of phages, and that the use of resistant strains may allow one to control the formation of a phage ecosystem at the production site [42]. CRISPR loci were identified in three strains. In this case, the absence of Cas proteins is consistent with data on incomplete or degraded CRISPR systems in Lactococcus [43].

Phage DNA sequences were found in the genomes of L. lactis 51n and 43n and L. cremoris 23. In 51n and 43n, these sequences likely correspond to extragenomic satellite phages or viruses that entered the samples during culturing. The prophage detected in L. cremoris 23 is likely integrated, but activation experiments are required to confirm its ability to be induced.

According to previously published data, L. cremoris generally exhibits a lower fermentation rate than L. lactis [44], although individual cremoris strains may exhibit higher activity than some lactis strains [8]. The decreased enzymatic activity observed in the L. lactis 51n and 43n and L. cremoris 23 strains compared with that in L. lactis 73n and 81n may also be due to intraspecific differences, the cause of which remains to be determined. Despite the lack of complete cell lysis, the presence of prophages in the genome likely creates additional physiological stress. This may be due to the activation of abortive defense systems (AbiC, AbiG, etc.) that prevent phage dissemination but simultaneously disrupt normal cellular metabolism, which may reduce the overall functional activity of the bacterial colony due to concomitant metabolic stress.

## CONCLUSION

This study demonstrated that the metabolic features of Lactococcus used in lactic acid fermentation are largely determined by strain-specific characteristics rather than phylogenetic affiliation. This is supported by the pronounced phenotypic variability of closely related L. lactis strains. The possible association between the presence of prophages and reduced metabolic activity in the L. lactis 51n and 43n and L. cremoris 23 strains stresses the importance of phage profiling upon selection of strains for industrial use. Furthermore, the presence of the AbiB system in 51n and 73n strains resistant to a wide range of bacteriophages makes this system a promising marker of phage resistance. Taken together, these findings emphasize the need for an integrated approach combining genomic and phenotypic methods to effectively select strains with high productivity and resistance for use in the dairy industry
